# In Memoriam: Scott M. Hammer, MD

**DOI:** 10.1002/jia2.25856

**Published:** 2021-12-08

**Authors:** Wafaa M. El‐Sadr, Judith S. Currier, Larry Corey

**Affiliations:** ^1^ ICAP at Columbia University and Department of Epidemiology and Medicine Columbia University New York City New York USA; ^2^ Division of Infectious Diseases David Geffen School of Medicine, UCLA, California Los Angeles USA; ^3^ Department of Medicine and Laboratory Medicine University of Washington Seattle Washington USA

1

The Infectious Disease community lost a giant, Dr. Scott M. Hammer, on 17 November 2021. Scott is widely acknowledged as having been a brilliant researcher, a gifted clinician and a devoted mentor. He was born in New York City and obtained his undergraduate and medical degrees at Columbia University. This was followed by training in internal medicine and infectious diseases at New York Presbyterian Hospital, Stanford University Hospital and Massachusetts General Hospital, where he trained with many of the titans of the Infectious Disease field. He spent his professional career first at the New England Deaconess Hospital in Boston, and then at Presbyterian Hospital in New York City and the Columbia University Irving Medical Center, taking on the position of Harold C. Neu Professor of Infectious Diseases and Professor of Epidemiology. He expanded the work of the Division of Infectious Diseases in his capacity as Division Chief, focusing on growing its research portfolio.

Scott's career began as the HIV epidemic in the United States unfolded and, in many ways, the epidemic shaped his career, which was true for many infectious disease specialists of that era. Few others, however, were able to shape the epidemic in the ways that Scott did. The suffering of so many patients compelled him to seek to identify effective treatments for HIV. He fully engaged with the U.S. NIH‐funded AIDS Clinical Trials Group and led pivotal studies that identified life‐saving antiretroviral regimens at a time when few options existed. His contributions to HIV virology, HIV resistance and the evaluation of virological and immunological markers among persons living with HIV on and off treatment furthered our understanding of the pathogenesis of HIV infection. When the major advances achieved by Scott and others allowed HIV to be transformed into a chronic and manageable condition, he turned his attention to the challenge of HIV prevention. Scott focused in particular on the quest for an HIV vaccine. He was tireless in the pursuit of this goal, and as a leader in the NIH‐funded HIV Vaccine Trials Network, he engaged deeply in the design and implementation of several critical vaccine studies.

Some use the expression “a doctor's doctor,” and Scott epitomized this phrase. His clinical acumen was legendary, never resting until he arrived at a diagnosis and a way forward for his patients. He genuinely cared for his patients, and this was palpable to all, particularly to them. His colleagues looked to him for his insights when they exhausted all options, when they were unable to reach a diagnosis or were seeking a way to reassure a patient. His ability to listen carefully and to pull all the strands of evidence together into a coherent whole was not only impressive to his colleagues, but also inspiring to students and trainees.

Medicine is in many ways an apprenticeship. While knowledge is critically important for the creation of skilled physicians, beyond that, the art of medicine is learned by observing mentors and emulating them. For generations of students and trainees, Scott was an incomparable teacher and mentor. He demonstrated compassion, wisdom, patience and respect in his every interaction, leaving an indelible mark on all those who were fortunate to be mentored by him over the years.

While our professional careers have intersected with Scott's over the past several decades with many wonderful memories of productive discussions and exciting work, we look back to the uniqueness of his contributions. Beyond his many stellar academic achievements, Scott's legacy is his overarching humanity. For all of us who knew Scott, his wisdom, kindness, humility and generosity were fundamentally what defined him. What better legacy can a person hope for!

## COMPETING INTERESTS

The authors have declared no competing interests.

## AUTHORS’ CONTRIBUTIONS

All authors have contributed to the preparation of the manuscript, read and approved the final draft.

